# Harnessing a multi-dimensional fibre laser using genetic wavefront shaping

**DOI:** 10.1038/s41377-020-00383-8

**Published:** 2020-08-26

**Authors:** Xiaoming Wei, Joseph C. Jing, Yuecheng Shen, Lihong V. Wang

**Affiliations:** 1 Caltech Optical Imaging Laboratory, Andrew and Peggy Cherng Department of Medical Engineering, Department of Electrical Engineering, California Institute of Technology, 1200 East California Boulevard Mail, Code 138-78, Pasadena, 91125 CA USA; 2 Present Address: School of Physics and Optoelectronics; State Key Laboratory of Luminescent Materials and Devices; Guangdong Engineering Technology Research and Development Center of Special Optical Fiber Materials and Devices; Guangdong Provincial Key Laboratory of Fiber Laser Materials and Applied Techniques, South China University of Technology, 381 Wushan Road, Guangzhou, 510640 China; 3 Present Address: School of Electronics and Information Technology, Sun Yat-sen University, Guangzhou, 510006 China

**Keywords:** Fibre lasers, Optical manipulation and tweezers

## Abstract

The multi-dimensional laser is a fascinating platform not only for the discovery and understanding of new higher-dimensional coherent lightwaves but also for the frontier study of the complex three-dimensional (3D) nonlinear dynamics and solitary waves widely involved in physics, chemistry, biology and materials science. Systemically controlling coherent lightwave oscillation in multi-dimensional lasers, however, is challenging and has largely been unexplored; yet, it is crucial for both designing 3D coherent light fields and unveiling any underlying nonlinear complexities. Here, for the first time, we genetically harness a multi-dimensional fibre laser using intracavity wavefront shaping technology such that versatile lasing characteristics can be manipulated. We demonstrate that the output power, mode profile, optical spectrum and mode-locking operation can be genetically optimized by appropriately designing the objective function of the genetic algorithm. It is anticipated that this genetic and systematic intracavity control technology for multi-dimensional lasers will be an important step for obtaining high-performance 3D lasing and presents many possibilities for exploring multi-dimensional nonlinear dynamics and solitary waves that may enable new applications.

## Intelligent 3D lasers: Bio-inspired algorithms help tame multimode fiber lasers

Multimode fibres that contain numerous groups of lightwaves, or modes, can now assist lasers in producing tunable emission characteristics. Lihong Wang from the California Institute of Technology in Pasadena, United States, and colleagues have developed a technique to control the complex interactions inside a multimode fibre laser using machine learning. The team created a feedback loop where portions of laser signals travelling through a multimode fibre are sent to a spatial light modulator that adjusts the phases of propagating waves. By using genetic algorithms to direct the spatial light modulator, this setup can optimize parameters such as output power after a few minutes of evolutionary feedback—changing, for instance, an initial speckle-shaped light pattern into a single focused spot. Other tunable laser features including wavelength scanning and pulse control were demonstrated with this approach.

## Introduction

Lightwave propagation in multimode fibres (MMFs) that support many transverse modes is gaining new interest for generating high-performance coherent three-dimensional (3D) lightwaves and exploring multi-dimensional nonlinear dynamics and solitary waves^1–5^. In MMFs, fruitful modal interaction, temporal randomization, and spectral evolution coexist and impose extra degrees of complexity for interdisciplinary studies. In particular, lightwave propagation in the nonlinear regime of MMFs can lead to various multi-dimensional nonlinear physics phenomena, such as soliton self-frequency shifting^6^, dispersive waves^7^, spatial beam self-cleaning^8^, intermodal nonlinear mixing^9^ and self-organized instability^10^. More recently, spatiotemporal mode-locking (STML) in an MMF laser, as a kind of multi-dimensional optical dissipative system^11^, has also been reported^12^, wherein many transverse and longitudinal modes are phase-locked together in three dimensions to generate self-organized femtosecond (fs) 3D pulses with complexly interfering spatial patterns. This novel concept opens up new opportunities for the generation of extremely high-energy fs pulses, the study of multi-dimensional nonlinear lightwave dynamics, and the engineering of spatiotemporal coherent light fields. Despite these fascinating opportunities, systematic control over a variety of coherent lightwave oscillations in these multi-dimensional optical systems, which is essential for the adoption of this novel technology by both mainstream applications and studies of multi-dimensional nonlinear dynamics in physics, chemistry, biology, and materials science, is still in its infancy. The design of control schemes for multi-dimensional oscillators is complicated and challenging since many factors, including chromatic dispersion, modal dispersion, nonlinearity, gain and loss, are nonlinearly coupled and contribute to complexity and uncertainty, thus imposing additional rigorous control requirements. In addition, directly applying traditional one-dimensional (1D) technologies to control multi-dimensional optical systems is not straightforward^13^.

The concept of wavefront shaping has been developed and extensively applied to manipulate scattered photons inside turbid media in three dimensions^14–16^. In general, wavefront shaping technologies treat optical scattering within the speckle correlation time as a deterministic process that has a fixed input–output relation^17–19^. To generate a desired optical field, the wavefront of a light field can be simply shaped using spatial light modulators (SLMs) before being sent into the scattering media. Motivated by its state-of-the-art abilities, the field of wavefront shaping has undergone rapid development and intensive studies in terms of biophotonics^17^, laser scanning^20^ and nonlinear physics^5^. Specifically, in multimode waveguides, the multiple spatial degrees of freedom cause a propagating lightwave to appear as if it has been highly scattered. Thus, wavefront shaping can provide an effective means to manipulate lightwave propagation in multimode waveguides, even under strong mode coupling conditions^5,21–23^. Prior works of wavefront shaping reported in either conventional scattering media or multimode waveguides have predominately focused on passive systems with input–output relationships that can be considered linear transformations, although an active amplifier system has recently been investigated^24^. The multi-dimensional optical oscillation system, on the other hand, operates in a much more complicated scenario, wherein spatiotemporal dispersion, nonlinearity, gain and loss can nonlinearly interact—breaking the linearity of the lightwave transmission within a waveguide. To date, no effort has been dedicated to controlling the nonlinear lightwave oscillation in multi-dimensional lasers, leaving this field largely unexplored with opportunities to exploit the multi-dimensional degrees of complexity for obtaining high-performance 3D lasers and studying multi-dimensional nonlinear dynamics and solitary waves.

In this work, we harness a multi-dimensional laser using genetic wavefront shaping technology. Specifically, the multi-dimensional laser is an emerging MMF laser that simultaneously supports many longitudinal and transverse modes. The underlying complexities are decomposed by genetically shaping the wavefront of a speckled lightwave that oscillates inside the multimode laser cavity. The performance in each dimension of the MMF laser can be intelligently manipulated through customization of the objective function (or cost function) of the genetic algorithm. As a result, we successfully harness automated control over optical power, spatial mode, operating wavelength, and mode-locking initiation of the MMF laser. We believe that our results could unlock new potentials for generating high-performance coherent 3D lasers and facilitate the discovery and understanding of new multi-dimensional coherent lightwaves, which in turn may inspire new applications.

## Results

**Fig. 1:**
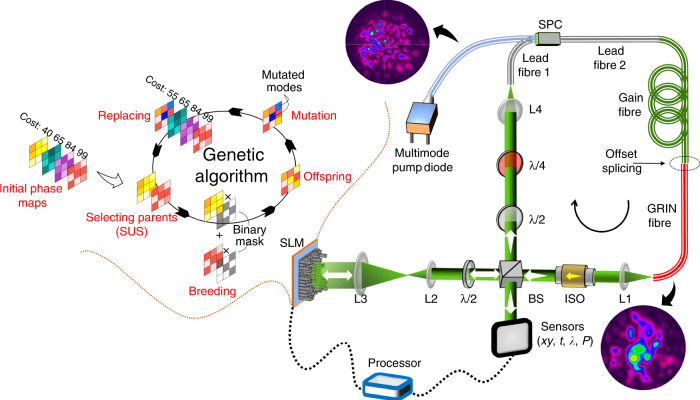
Schematic diagram of a genetic multi-dimensional fibre laser using wavefront shaping. A multimode gain fibre (Thorlabs YB1200-25/250DC) is pumped through a signal–pump combiner (SPC) by a multimode pump laser diode (976 nm wavelength, 27 W maximum power). The SPC has lead fibres (i.e., lead fibres 1 and 2, CorActive DCF-UN-25/250-08) with core/cladding diameters of 25/250 μm. The output of the GRIN fibre is collimated by a lens (L1), whose state of polarization (SOP) is subsequently linearized by a polarization-dependent isolator (ISO). A portion of the laser beam (signal) is extracted by a cubic beam splitter (BS), while the rest is expanded by a telescope (L2 and L3, 5× magnification). The magnified laser beam is fed into a spatial light modulator (SLM) for wavefront control. To obtain an optimal wavefront modulation efficiency, the SOP of the laser beam is adjusted by a half-wave plate (λ/2, next to L2). The return laser beam is coupled back into the multimode fibre (i.e., lead fibre 1 of the SPC in this case) by L4. Another half-wave plate and a quarter-wave plate (λ/4) are utilized to control the SOP of the incident laser beam being amplified. Insets show the typical spatial mode profiles of the pump and signal laser beams. The extracted signal is detected by multiple sensors in different domains, including the cross-section space *xy*, time *t*, optical wavelength *λ*, and optical power *P*. The outputs of the sensors are processed by a customized genetic algorithm to create a phase map (see Fig. S1), which is then fed into the SLM

Figure 1 presents a schematic diagram of a genetic MMF laser leveraging wavefront shaping. The MMF laser can be three-dimensionally mode-locked through STML with strong spatial and spectral filtering in addition to high nonlinearity, gain and spatiotemporal dispersion^12^, wherein ultrashort pulse generation is initiated and stabilized by nonlinear polarization evolution (NPE)^25^. In brief, the MMF laser has a ring cavity, where the laser beam propagates clockwise. The gain medium is a piece of double-cladding ytterbium-doped MMF (Thorlabs YB1200-25/250DC, ~3 m long) that supports approximately 13 modes at 1060 nm (Table S1). This multimode gain fibre has a core diameter of ~25 µm, a core/cladding numerical aperture (NA) of 0.07/0.48, and a cladding-pump absorption of 2.3 dB/m at 920 nm. Using a cladding-pump scheme, the multimode gain fibre in an optical amplifier configuration can provide a slope efficiency of >75%. The laser system is pumped by a 976-nm multimode laser diode (27 W maximum power) through a signal–pump combiner (SPC). The lead fibres of the SPC (CorActive DCF-UN-25/250-08), which have core/cladding diameters of 25/250 μm, are designed to match with the gain fibre. A piece of the multimode graded-index (GRIN) fibre (Thorlabs GIF625, 62.5 µm core diameter, ~1 m long) is fusion-spliced to the gain fibre with a core offset (approximately 20 µm, as indicated in Fig. 1) to excite the higher-order modes. Please note that the GRIN fibre, which is designed with reduced modal dispersion of a magnitude comparable to its chromatic dispersion, can support hundreds of transverse modes and is key for successful STML^12^.

The output of the GRIN fibre is collimated by a lens (L1) and launched into free space. A polarization-maintaining isolator (ISO) is utilized to establish a polarization-dependent transmission mechanism that is associated with the intensity-dependent fibre nonlinearities for NPE. A 50:50 beam splitter (BS) serves as an output coupler and extracts half of the laser beam for characterization. A portion of the laser beam from the BS is launched to the wavefront shaping unit, which consists of a half-wave plate (λ/2), a 5× telescope (lenses L2 and L3) and a fast phase-only SLM (Holoeye PLUTO-2-NIR, 1920 × 1080 pixels, 60 Hz frame rate). The half-wave plate (λ/2) is used to optimize the state of polarization (SOP) of the laser beam and thus improve the modulation efficiency of the SLM, while the telescope magnifies the laser beam to match with the active area of the SLM. The modulated laser beam is returned and subsequently coupled back into the MMF (i.e., lead fibre 1 of the SPC) through the BS, wave plates (λ/2 and λ/4) and L4. The SOP of the laser beam is further adjusted by a half-wave plate (λ/2) and a quarter-wave plate (λ/4). The extracted laser beam from the BS is detected in multiple domains, including cross-section space *xy*, time *t*, optical wavelength *λ*, and optical power *P* (via a CCD camera, photodiode, spectrometer, and power metre, respectively; see Table S2). The detected multi-dimensional signals serve as the cost values of the corresponding cost functions and are fed to the genetic algorithm for evaluating and generating the phase maps (see “Methods”).

**Fig. 2:**
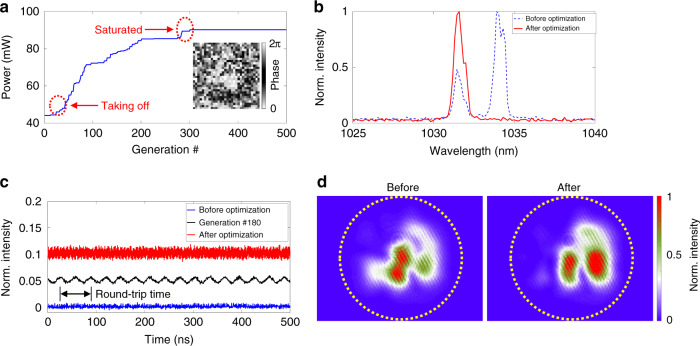
Optical power enhancement of the genetic multi-dimensional fibre laser working in the quasi-CW state. **a** Genetic power enhancement. The inset shows the optimal phase map displayed on the SLM. Please note that the optical power has been attenuated by 10 dB to avoid saturating the power metre. **b** Optical spectra before and after optical power enhancement. **c** Real-time output signal in the temporal domain. Here, the curves have been vertically offset for better visualization. **d** Mode profiles before and after optical power enhancement. The dotted circles indicate the core-cladding boundary

**Fig. 3:**
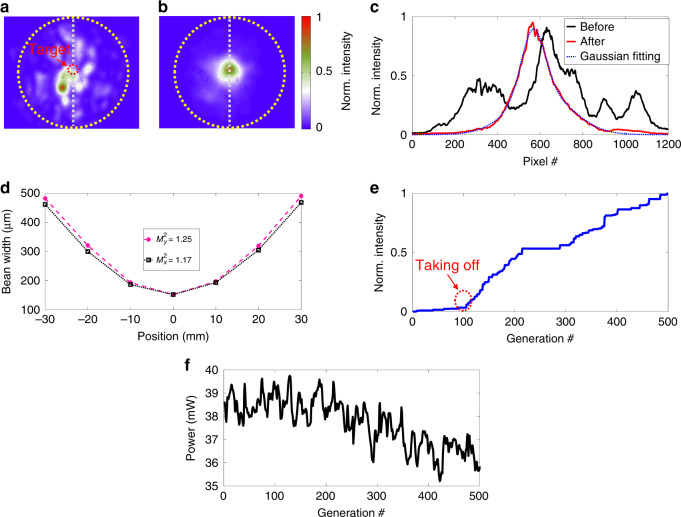
Mode profile cleaning of the genetic multi-dimensional fibre laser working in the quasi-CW state. **a** Mode profile before genetic optimization, recorded by a CCD camera. The dotted circles indicate the core-cladding boundary. **b** Mode profile after genetic optimization. **c** Line profiles of the mode patterns, as indicated in (**a**) and (**b**). **d** *M*^2^ measurements for the *x* and *y* directions. **e** Intensity evolution of the targeted area selected for genetic optimization, as indicated by the red dotted circle in (**a**). **f** Corresponding optical power evolution during the mode profile optimization. Here, the optical power is attenuated by 10 dB

**Fig. 4:**
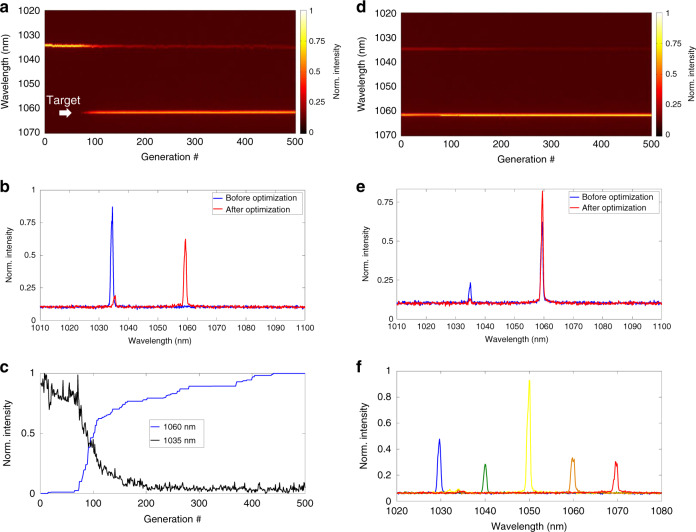
Wavelength manipulation of the genetic multi-dimensional fibre laser working in the quasi-CW state. **a** Evolution of the genetic wavelength switching. The white arrow indicates the targeted wavelength being switched to. **b** Optical spectra before and after wavelength switching. **c** Intensity evolution at wavelengths of 1035 nm and 1060 nm. **d** Evolution of the genetic side mode suppression ratio (SMSR) improvement. **e** Optical spectra before and after SMSR improvement. **f** Genetic wavelength tuning

**Fig. 5:**
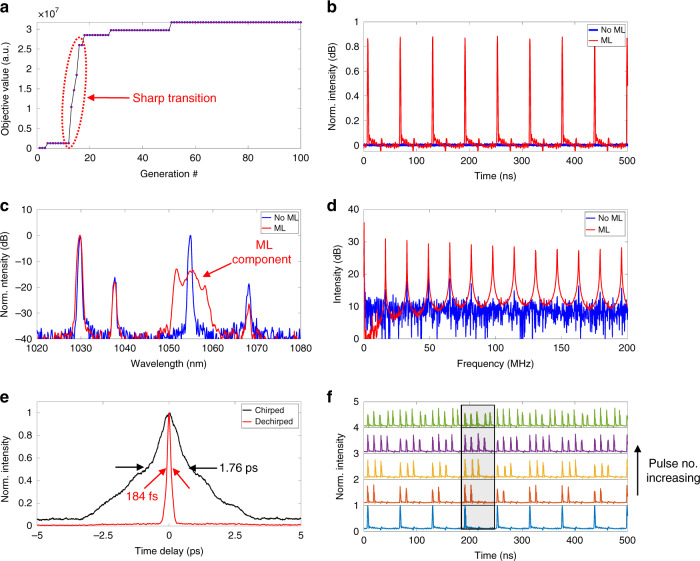
Mode-locking manipulation of the genetic multi-dimensional fibre laser. **a** Evolution of the cost function value during genetic mode-locking. **b** Temporal signals before and after mode-locking. **c** Optical spectra without and with mode-locking. Here, only one of the CW wavelength components has evolved into the mode-locking state. **d** RF signals before and after mode-locking, calculated from the temporal signals using a fast Fourier transformation. **e** Autocorrelation traces of the chirped and dechirped pulses. **f** Generation of multiple pulses. The pulse trains are vertically offset for better visualization

In general, this MMF laser can operate in the STML state by increasing the pump power beyond 15 W, together with an appropriate setting of the SOP^12^. The output optical power can be varied from tens to hundreds of mW, which depends on the SOP of the laser. The laser cavity has a fundamental repetition rate (FRR) of ~16 MHz (i.e., a round-trip time of ~61 ns). The insets of Fig. 1 show the typical mode profiles of the pump and signal beams. In the following experiments, we first investigate the optical power, mode profile, and lasing wavelength of the MMF laser in the quasi-CW state without specific optimization for mode-locking, i.e., Figs. 2–4, respectively. Please note that here, the quasi-CW state of the MMF laser refers to the operating condition without stable mode-locking but does not exclude the occasional generation of broad-pulse fluctuations (or modulations) due to random mode-beating. Then, we study the pulsing ability of the MMF laser through automatic mode-locking manipulation, i.e., Fig. 5.

Lightwaves propagating in MMFs can create complex 3D interference patterns (insets of Fig. 1). Thus, once lasing is established, the interfering lightwaves in all modes are amplified simultaneously, and complex combinations of laser modes (both transverse and longitudinal modes) compete for the limited energy available from the gain medium. As a result, the speckled laser beam experiences uneven gain in both the transverse and longitudinal dimensions, which can compromise the overall optical power. Here, the output optical power of the MMF laser working in the quasi-CW state is optimized by genetically evaluating the cost function *P*_average_ using the reading from a power metre with an attenuation of 10 dB. Please note that all the measurements of the output optical power shown in this work are 10 dB attenuated. The optimization starts from a cavity setting where the MMF laser is adjusted to deliver the “highest” power by only rotating the wave plates. As shown in Fig. 2a, the output optical power can typically be increased by a factor of approximately 2 after approximately 300 generations of breeding, which takes approximately 4 mins. As can be observed, the genetic wavefront shaping exhibits reasonably high sensitivity, and the output power becomes >5% above the baseline after ~30 generations of breeding. The power enhancement gradually becomes saturated after 300 generations of breeding, after which the relative change is less than 1%. Once the optical power is saturated, the phase maps of the population become closely consistent, and the phase maps exhibit a high overall cross-correlation, i.e., increasing from 0% to ~75%. Figure 2b, c shows the optical spectra and temporal signals before and after optical power optimization. Surprisingly, the optical spectrum was cleaned after genetic optimization, i.e., changing from dual bands to a single band, while both temporal signals exhibit noisy baselines without sharp pulses, which is expected since no specific optimization is present to promote mode-locking; rather, that condition is demonstrated in Fig. 5. These results also indicate that the initial lasing condition favours a multiwavelength operation, which can arise from either the alleviation of gain competition through intensity-dependent nonlinearity (i.e., the Kerr effect)^26^ or nonlinear coupling between the transverse modes. Figure 2d shows the mode profiles before and after the optical power optimization. As can be observed, the mode profile is slightly changed after the optical power optimization, which, to a certain extent, is expected since here the objective function is set only to optimize the optical power such that the mode profile can vary relatively freely.

The larger mode area of MMFs, on the one hand, can deliver a much greater optical power and mitigate the nonlinear effect, which sets the ultimate limit on the ultrashort pulse propagation; on the other hand, the increased mode area creates much weaker confinement in the transverse space and leads to the coexistence of multiple transverse modes, easily up to hundreds. As a result of the strong nonlinear coupling and interference among these modes, the quality of the mode profile in MMF systems is typically worse than that of the single-mode counterparts—usually evaluated by the *M*^2^ factor^27^. Recently, Teğin et al. demonstrated an MMF laser with *M*^2^ < 1.4 through spatiotemporal self-similar evolution^28^, and Krupa et al. presented laser beam cleaning in the nonlinear propagation regime of the GRIN fibre^8^. In general, a laser with poor (spatial) beam quality is not suitable for applications requiring diffraction-limited focusing, e.g., precision machining, high-resolution optical microscopy, and long-range metrology. To genetically improve the beam quality of a MMF laser, we set the objective function to the mean intensity of the targeted area, i.e., 〈*I*(*x*_0_,*y*_0_)〉, as indicated by the red dotted circle in Fig. 3a, which is measured by a CCD camera (Point Grey GS3-U3-32S4M-C). The mode profile cleaning starts from a mode profile with a random phase map addressed to the SLM. Figure 3a shows the mode profile before genetic optimization, which comprises a complex speckled pattern caused by the coexistence of many transverse modes. The optimized mode profile, fascinatingly, is much cleaner and appears as a single bright spot (Fig. 3b**)**. The final mode pattern exhibits a Gaussian-like profile with a weak background of higher-order modes (Fig. 3c). *M*^2^ measurements are conducted to evaluate the beam quality after optimization. As shown in Fig. 3d, *M*^2^ factors of 1.17 and 1.25 are obtained for the *x* and *y* directions, respectively. The evolution of the objective value, as shown in Fig. 3e, demonstrates that the optimization of the mode profile is less sensitive than that of the optical power, and obvious intensity enhancement (by 5%) of the targeted area occurs only after ~100 generations of breeding. It is worth noting that spatiotemporal self-similar evolution^28^ and Kerr nonlinear cleaning^8^ have also been demonstrated as powerful approaches for improving the beam quality in MMF systems. Here, instead, cleaning of the mode profile in the MMF laser using wavefront shaping leads to the generation of a high-quality beam in a genetic manner. As such, a highly MMF laser can also deliver a high-quality laser beam without compromising the optical power, as shown in Fig. 3f, where the optical power exhibits likely random variation at approximately 37 mW (10 dB attenuated) with a standard deviation of ~1.1 mW.

Wavelength tunability is commonly required in different research fields, including optical component testing, fibre optic sensing, metrology, and optical spectroscopy^29^. To this end, we manipulate the optical spectrum of the MMF laser using genetic wavefront shaping technology. As shown in Fig. 4a, the initial lasing wavelength is approximately 1035 nm. The cost function of the genetic algorithm is evaluated by the intensity at the targeted wavelength, i.e., *I*(*λ*_target_), which is measured by a spectrometer (Avantes AvaSpec-ULS3648). Please also note that the targeted wavelength can be different from or the same as the initial wavelength, while the latter case is equivalent to optimizing the optical power. We first demonstrate wavelength switching, i.e., from the initial wavelength to another optical wavelength (1060 nm in this case); see Fig. 4a, b. As shown, the optical spectrum begins to oscillate at the targeted wavelength after ~90 generations of genetic iteration. Once lasing at the targeted wavelength is established, the intensity at the original wavelength gradually decreases (Fig. 4c). Figure 4b shows the line plots of the initial and final optical spectra after optimization and clearly illustrates that a new wavelength at ~1060 nm has been established from the noise floor where no initial signal is detected prior to running the genetic algorithm, as shown in Fig. 4c.

However, the optimized optical spectrum has a residual component at the initial wavelength, resulting in a poor side mode suppression ratio (SMSR) of ~10 dB (Fig. 4b). Some specific applications require a clean optical spectrum, as unwanted wavelength components can become noise sources. Thus, strong side mode suppression is highly desired. To this end, the objective function is modified from a simple form of *I*(*λ*_target_) to *I*(*λ*_target_)/*I*(*λ*_initial_). As shown in Fig. 4d, the initial wavelength component has indeed been further attenuated, and thus, the SMSR is significantly improved. Figure 4e illustrates the optical spectra before and after enhancement of the SMSR, and an SMSR improvement of 5.6 dB is obtained. Notably, this wavelength switching capability can be further applied to perform wide range wavelength scanning. As shown in Fig. 4f, wavelength scanning can cover the entire operational bandwidth of ytterbium-doped gain media, typically from 1030 to 1070 nm. It is worth noting that the intensities of optical spectra vary across the scanning range, which can be a potential issue for applications requiring superior power stability. This weakness can potentially be mitigated by using power equalization technologies^30^. Please also note that the speed of wavelength scanning in the current setup is generally slow (tens of mins). If necessary, however, emerging high-speed wavelength wavefront shaping technologies^31^ can be employed to accelerate the speed.

Finally, the pulsing ability of the MMF laser is investigated using genetic wavefront shaping. It is also worth noting that automatic mode-locking has been intensively studied for single-transverse-mode fibre lasers^32–36^, while no effort has so far been devoted to MMF lasers—mainly owing to their multi-dimensional complexities, which make directly applying the traditional single-transverse-mode (1D) technologies challenging. In this case, the objective function of the genetic wavefront shaping is evaluated using the radiofrequency (RF) spectrum. In brief, the temporal signal is first detected by a photodiode (PD) and is subsequently digitized by a data acquisition (DAQ) card. The recorded digital temporal signal is converted to the frequency domain using a fast Fourier transformation (FFT), resulting in the RF spectrum of the temporal signal. Because the FRR of the MMF laser can be calculated simply from the total cavity length, the RF intensities of the FRR frequency and its higher-order harmonics, typically up to tens of orders (limited to the electronic bandwidth), are obtained. In this case, the objective function of automatic mode-locking is evaluated by measuring the RF intensity of the 10th harmonic *I*(*f*_10th_). Please note that other alternative objective functions can also be applied, e.g., the intensity of the FRR frequency or the sum of the intensities of all harmonics. However, our experimental study suggests that the case of the intensity of the FRR frequency provides a worse performance for successful automatic mode-locking. This may have been influenced by the weak pulsed signal generated by the beating/interference between the free-running longitudinal modes, which thus increases the failure rate of automatic mode-locking.

In our experiments, automatic mode-locking using genetic wavefront shaping starts from an initial condition without any detectable pulses, which is realized by randomizing the wave plates of the laser cavity to set the laser out of the mode-locking condition. Figure 5a shows the evolution of the objective value during automatic mode-locking using genetic wavefront shaping. Different from previous cases where the evolutions of the objective value are gradually changed, here, the system exhibits a sharp transition within only a few generations of breeding such that the objective value increases by ~20 times from generation 12 to 16. This occurs mainly because the system parameters that can enable STML have a wide range of values, and an approximate wavefront can initiate a strong pulse that subsequently triggers self-evolved mode-locking, as also shown in previous 1D studies^33^. During the mode-locking manipulation, the mode profile exhibits variation similar to the optical power variation in the case of mode profile cleaning (Fig. S2a, b). However, the mode profile becomes steady once the genetic optimization is finished (Fig. S2c). Figure 5b depicts the temporal signals before and after mode-locking, wherein a pulse train is clearly demonstrated after successful automatic mode-locking. The pulse period is approximately 61 ns, corresponding to an FRR of ~16 MHz. Before mode-locking, the optical spectrum, as shown in Fig. 5c, manifests a complicated scene in which multiple quasi-CW wavelength components coexist. Once mode-locking is established, the third wavelength component from the left is significantly broadened, while the other three quasi-CW components remain unchanged except for intensity variations. It is also noted that other cases involving only one wavelength component have been observed (Fig. S3). The spectral width of the mode-locked component is approximately 8 nm, corresponding to a transform-limited pulsewidth of approximately 150 fs. The pulsewidths before and after dechirping are 1.76 ps and 184 fs, respectively, as shown in Fig. 5e. It is worth pointing out that the mode-locking wavelength is especially sensitive to the initial setting of the wave plates, i.e., the linear polarization offset of the optical system.

Considering the nonlinear phase shift that accumulates in optical fibres, particularly when delivering intense ultrashort pulses with extremely high peak powers, the generation of multiple pulses coexisting in the same round trip is observed. To manipulate the evolution of the multiple pulses, we demonstrate the precise control of the number of coexisting pulses in an MMF laser. To this end, the objective function is first evaluated by the RF intensity of the 10th harmonic at a pump power of approximately 20 W, which supports the generation of multiple pulses. Once multiple pulses are obtained, the pump power is gradually decreased. As shown in Fig. 5f, different numbers of coexisting pulses are generated, i.e., one to five pulses. Please note that the maximum number of coexisting pulses is mainly determined by the pump power level, which can be attributed to the energy quantization effect of dissipative optical solitons^37–39^. One of the potential applications of multi-pulse mode-locking is the generation of high-repetition-rate pulses through harmonic mode-locking. Andral et al.^40^ successfully demonstrated automatically harmonic mode-locking in a single-mode fibre laser by incorporating a merit function with a combination of RF harmonics. They also proposed using the pump power as an additional gene and considered the effect of laser hysteresis.

## Discussion

To conclude, we have demonstrated an intelligent multi-dimensional optical system leveraging genetic wavefront shaping technology. The multi-dimensional complexities of the optical system have been decomposed such that the individual performance metrics can be genetically manipulated. The genetic optimization in this complicated optical system holds the following potential benefits: (1) The increased optical power (by a factor of ~2) can further strengthen the ability of high-energy fs pulse generation using MMFs, in addition to their nature of handling higher power in a larger mode area. (2) The genetic cleaning of the mode pattern, from a speckled laser beam to a clean one, can benefit a wide spectrum of applications, such as high-resolution microscopy, high-throughput spectroscopy, and nanoscale machining, all of which require diffraction-limited focusing. Together with increased optical power, this genetic pulsed laser provides extraordinarily high brightness (or radiance) at the focus spot. (3) Wavelength switching and scanning are essential for optical sensing, metrology, and spectroscopy. In laser systems delivering high-energy ultrashort pulses, wavelength tuning using standard optical filtering is much more challenging than those operating in the low power regime (e.g., single-mode fibre systems), as the standard optical filters (either absorptive or interference coating ones) have limited operating wavelength ranges, as well as a set damage power threshold. (4) As demonstrated in prior works by Wright et al.^12^, Qin et al.^41^, Teğin et al.^28^, etc., STML is usually self-stated with the appropriate setting of the SOP and pump power. Here, genetic manipulation can potentially further enhance the performance of MMF lasers in rigorous environments and can be an effective technology to stabilize the multimode optical system against perturbation. Not limited to MMF lasers, this technology can potentially be extended to other multi-dimensional oscillators built with rod-like fibres, thin disks, and other kinds of solid-state media.

In the current experimental setup, all controls of different performance metrics are realized by a single wavefront shaping unit in the spatial domain, which actually sets certain limits on further strengthening the performance metrics of the genetic MMF laser, as the manageable degrees of freedom are confined to phase-only wavefront shaping in the spatial domain. To offer extra degrees of freedom, wavefront shaping systems simultaneously employing phase, amplitude, and polarization modulations can be used. Furthermore, this complete genetic control does not have to be limited to the spatial domain and, in principle, can be extended to the spectral domain through spectral diffractors (e.g., diffraction gratings)^34,42^. The latency of the system can also be potentially reduced by either using advanced wavefront shaping technologies, particularly the emerging machine and deep learning technologies^43–46^, or replacing the standard SLMs with high-speed ones^31^, which have been proved to provide at least two orders of magnitude higher speeds. Finally, wavefront shaping is also a promising technology for ultrashort pulse compression through dispersion compensation^47^, which can thus be explored for multimode mode-locking to directly generate transform-limited pulses.

## Materials and methods

### Genetic wavefront shaping algorithm

The iteration of genetic wavefront shaping is performed by a customized MATLAB program that leverages a genetic algorithm, which has been intensively applied in various fields, while among many other applications, wavefront shaping is an emerging appellation. In brief, the genetic algorithm is modified from a previous work^48^, as sketched in Fig. S1. First, an initial population of 100 phase maps is created. Each phase map has a pixel number of 30 × 30. Please note that each pixel of the phase map is a binned group of SLM pixels, and their initial phase values are randomly assigned. During the iteration, the objective value of a specific cost function (space, time, wavelength, or power) is measured for each phase map. Then, the phase maps are ranked according to their objective values, with a higher objective value receiving a higher ranking. Subsequently, the program iteratively optimizes the phase maps through breeding and mutating. To this end, an arbitrary 30 × 30 binary mask *M* is generated for breeding. Two parent masks, *M*_m_ and *M*_p_, are randomly selected through a stochastic universal sampling method from the population in each generation, for which the higher-ranked phase map has a higher probability of being selected. The selected parent masks are then combined using *M*, i.e., *M*_new_ = *M*_m_*M*+*M*_p_(1−*M*). Some pixels of this new mask (offspring) are selected and mutated by randomly changing the phases. To avoid mutating too many optimized pixels of the phase map, the percentage of pixels being mutated decreases with the iteration, in which way it is easier to approach the optimal phase map (without overshooting). The objective values of the new phase maps are measured for the selected cost function, which is used to rank and replace the worst phase maps. In each generation, a number of new phase maps *G* are generated and used to replace a corresponding number of lowest-ranked phase maps of the existing population. The whole algorithm is operated through the MATLAB GUI (Fig. S4).

## Supplementary information

Supplemental Material
