# Supplementary material for: Harnessing a multi-dimensional fibre laser using genetic wavefront shaping
Source: Light Sci Appl. 2020 Aug 26;9:149. doi: 10.1038/s41377-020-00383-8 (PMC7450085; doi:10.1038/s41377-020-00383-8)
Supplement: Supplementary file 1 — Supplemental Material [file 41377_2020_383_MOESM1_ESM.docx]

**Supplementary Information for**

**Harnessing a multi-dimensional fibre laser using genetic wavefront shaping**

Xiaoming Wei^1,2^, Joseph C. Jing^1^, Yuecheng Shen^1,3^, and Lihong V. Wang^1,*^

^1^Caltech Optical Imaging Laboratory, Andrew and Peggy Cherng Department of Medical Engineering, Department of Electrical Engineering, California Institute of Technology, 1200 East California Boulevard, Mail Code 138-78, Pasadena, California 91125, USA

^2^Present address: School of Physics and Optoelectronics; State Key Laboratory of Luminescent Materials and Devices; Guangdong Engineering Technology Research and Development Center of Special Optical Fiber Materials and Devices; Guangdong Provincial Key Laboratory of Fiber Laser Materials and Applied Techniques, South China University of Technology, 381 Wushan Road, Guangzhou, 510640, China

^3^Present address: School of Electronics and Information Technology, Sun Yat-sen University, Guangzhou 510006, China

^*^Correspondence should be addressed to L.V.W. (LVW@caltech.edu)

1. **Supplementary figures**


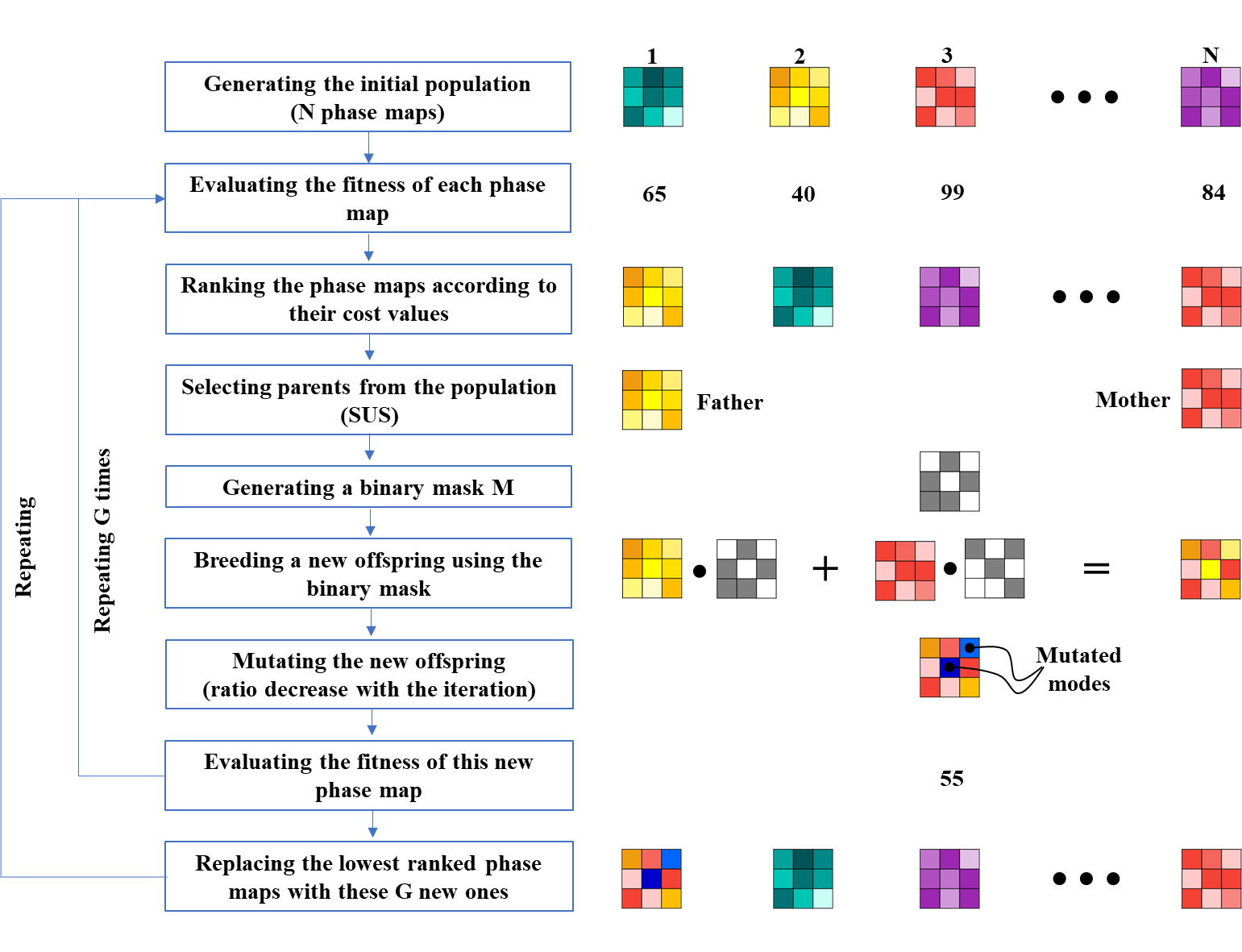


**Figure S1 |** **Flowchart of the genetic algorithm showing the steps**.


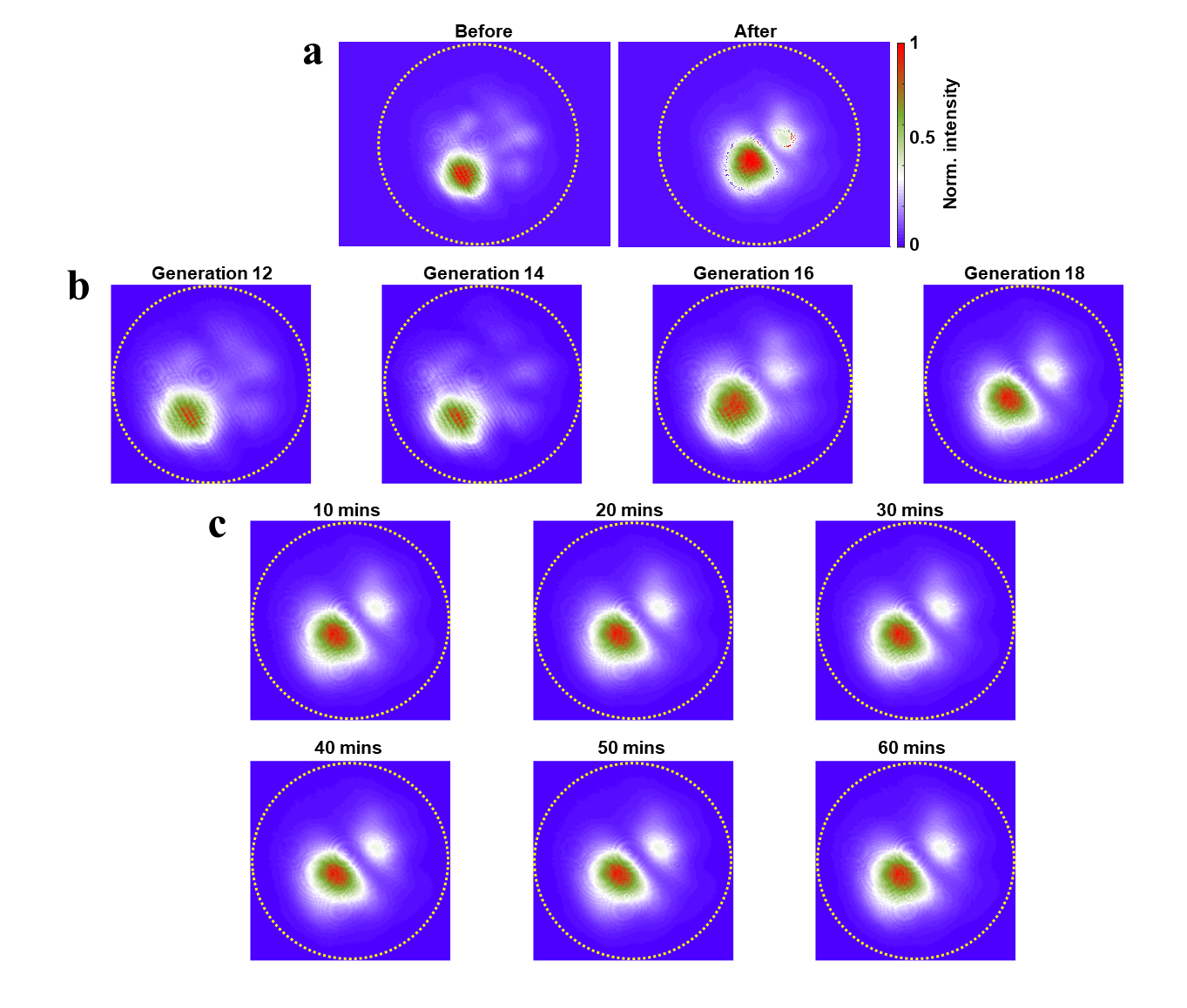


**Figure S2 |** **Mode profile evolution of the mode-locking manipulation**. **a**. Mode profiles before and after mode-locking manipulation. **b**. Evolution of the mode profile during the transition to mode-locking, i.e., from generation 12 to 18. **c**. Evolution of the mode profile after successful mode-locking. The dotted circles indicate the core-cladding boundary.


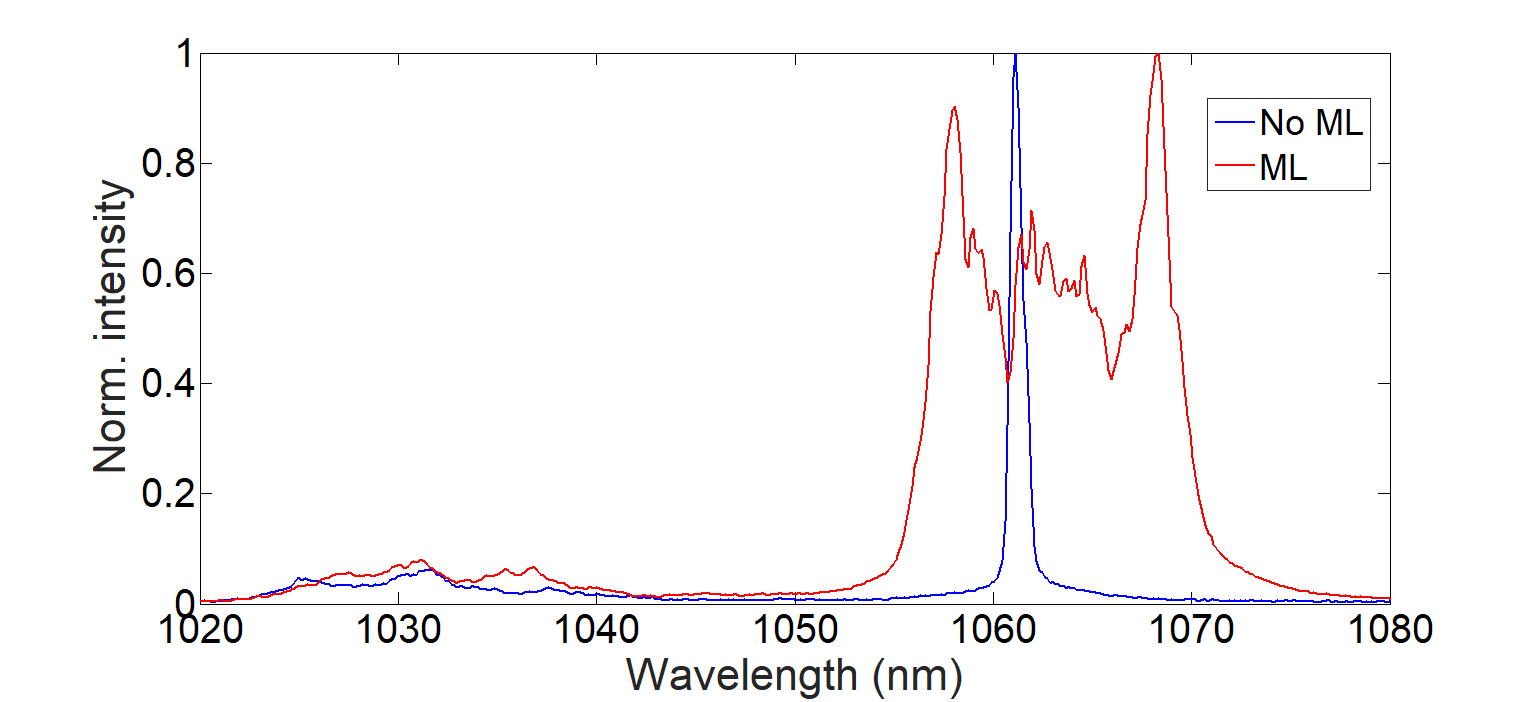


**Figure S3 |** **Mode-locking manipulation of the genetic multi-dimensional dissipative laser.** In this case, only one wavelength component is participated.


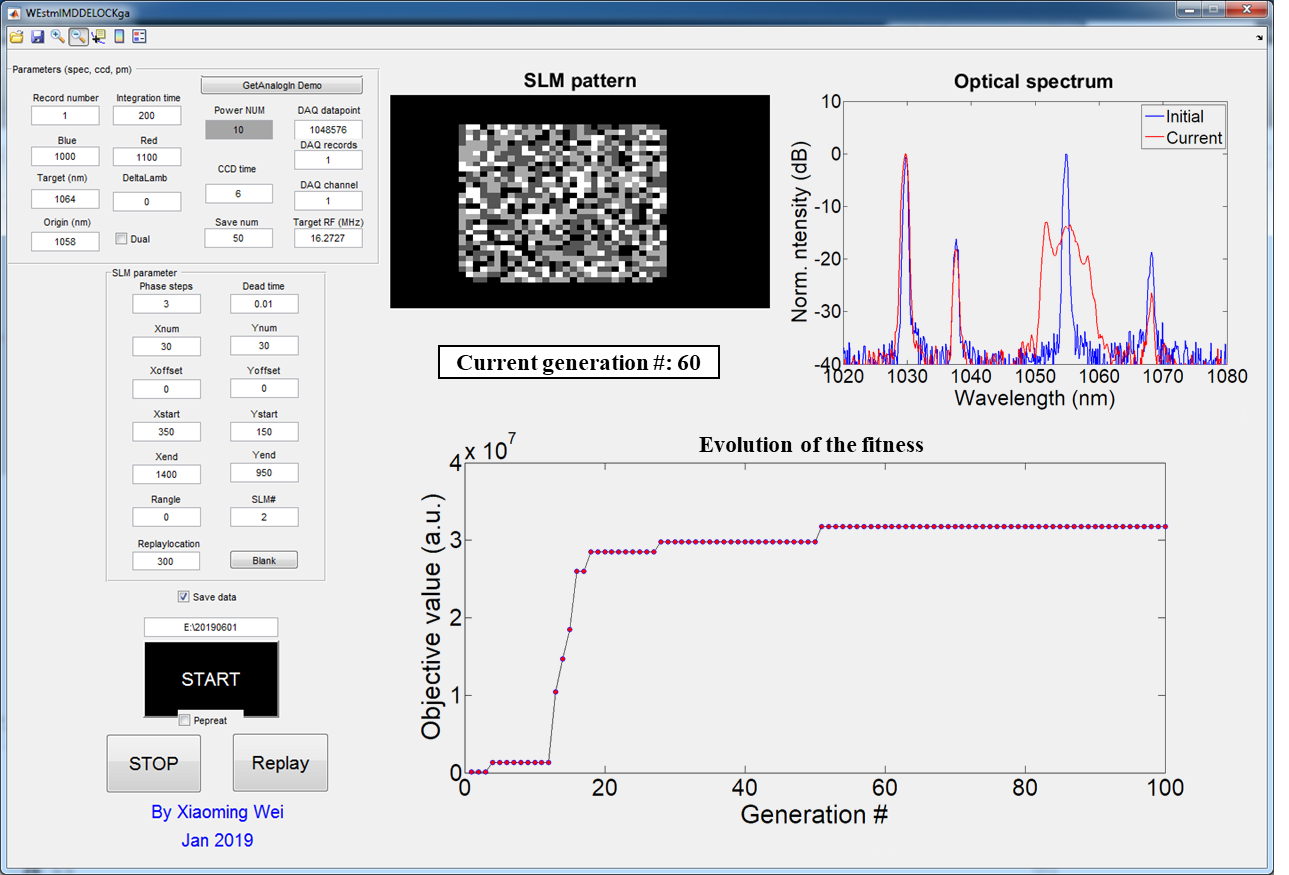


**Figure S4 |** **Customized co****ntrol program written in Matlab for genetic mode-locking**.

| **Table S1 Estimated numbers of modes of the fibers used in the multimode fiber laser** | | | | | |
| --- | --- | --- | --- | --- | --- |
| **Fiber** | **Model number** | **Core NA** | **Core radius/mm** | **V number** | **Mode no.^*^** |
| Lead fiber 1 | CorActive DCF-UN-25/250-08 | 0.08 | 12.5 | 5.93 | 18^†^ |
| Lead fiber 2 | CorActive DCF-UN-25/250-08 | 0.08 | 12.5 | 5.93 | 18^†^ |
| Gain fiber | Thorlabs YB1200-25/250DC | 0.07 | 12.5 | 5.19 | 13^†^ |
| GRIN fiber | Thorlabs GIF625 | 0.275 | 31.25 | 51.0 | 650^‡^ |
| **^*^**Counting the polarization; **^†^**Mode no. $\approx$ V^2^/2; **^‡^**Mode no. $\approx$ V^2^/4 | | | | | |

**Table S2 Key devices used in this study**

| **Device** | **Model no.** | **Function** | **Key parameters** |
| --- | --- | --- | --- |
| SLM | Holoeye PLUTO-2-NIR | Manipulating the phase of the laser beam | 1920 × 1080 pixels, 8.0 μm pixel size, 60 Hz frame rate |
| Camera | Point Grey GS3-U3-32S4M-C | Acquiring the mode profile | 2048 × 1536 pixels, 3.45 μm pixel size, 121 Hz frame rate, ~70% quantum efficiency at 532 nm. |
| Photodiode | EOT ET-3000A | Converting the optical signal to electronic one in the temporal domain | 400 ps rise time, 30 kHz – 1.5 GHz bandwidth, 1000 – 1600 nm wavelength range. |
| Spectrometer | Avantes AvaSpec-ULS3648 | Measuring the optical spectrum | 600 – 1100 nm wavelength range, ~0.5 nm resolution, 3.7 ms/scan acquisition speed |
| Power meter* | Thorlabs S121C | Measuring the average optical power | 400 – 1100 nm wavelength range, 10 nW resolution, 500 mW power limit, 9.7 × 9.7 mm^2^ active area |
| Digitizer | AlazarTech ATS9360 | Recording the signal in the temporal domain | 800 MHz bandwidth, 1.8 GS/s sampling rate, 12 bits resolution. |
| Oscilloscope | Rigol MSO/DS4000 | Recording the signal in the temporal domain | 500 MHz bandwidth, 4 GS/s sampling rate, 4 channels |
| **^*^**A 10 dB optical attenuator is added, i.e., a neutral density filter (Thorlabs ND510A) | | | |
